# Anti-aging medicine

**Published:** 2008-10

**Authors:** Balvant P. Arora

**Affiliations:** Horizon Plastic and Cosmetic Surgery, Mary Washington Hospital, Fredericksburg, VA 22401, USA

Today's healthcare challenges and tomorrow's opportunity can only be met by those who search out deeper explanations of the body processes that generate health and disease. Life expectancy has increased due to advances in medical science. However it has come with little progress towards quality of life or the length of disease-free years in the majority of population.

Most researchers believe that maximum life span in human is slightly over 110 years. Beyond that age, the estimates and speculation enter the realms of science fiction.

## GROWTH, DEVELOPMENT AND AGING

*Old age is the most unexpected of all things that happens to man*. -

Leon Trotsky

Aging has been a fact of life ever since it was created. Human beings go through various phases of life from being child to youth to being adult with youth being the best part of life from health point of view. Good health, strong muscles, an efficient immune system, a sharp memory and a healthy brain are characteristic of ideal youth. The hormones work at their peak capacity during the youth years.

Anti-Aging medicine aims to maintain or achieve this irrespective of chronological age i.e. to stay healthy and biologically efficient.

The prestigious scientific journal, Biogerontology, defines aging as: “The progressive failing ability of the body's own intrinsic and genetic powers to defend, maintain and repair itself in order to keep working efficiently.”

We are now living in the information age. Medical knowledge is increasing at an amazing rate-doubling every three years. This doubling rate of information is progressively decreasing. The world is changing and so is the way we view our health and well being as we age.

Aging has been believed to be inherent, universal, progressive natural phenomenon. It is detrimental with no benefits except perhaps wisdom. But now there is a paradigm shift in looking at the aging process based on firmly documented evidence in medical and scientific literature. If we plot the health in y-axis and the number of years in x-axis, the curve of life is like a triangle which is skewed with its apex at 25-30 years. Anti-aging helps to make it rectangle.

Many natural aging mechanisms frequently result in actual diseases. From this we can conclude that fighting an aging process may well bring about an improvement of an age related illness.

All animals or plants eventually show signs of aging even if they are in a protected environment. When we look at Ayurveda which prescribes plants or their components and extracts to fight against illness, one has to wonder why? Nature created these plant chemicals to help them protect against diseases or aging. Humans have later adapted these chemicals for their own use to increase their chances of maintaining their health.

### What is anti-aging medicine?

Anti-aging medicine is an evolving branch of medical science and applied medicine. It treats the underlying causes of aging and aims at alleviating any age related ailment. Its goal is to extend the healthy lifespan of humans having youthful characteristics.

Conventional and alternative medical disciplines are used in an integrated approach to achieve the best possible result for the patient. It is a holistic discipline, seeing the patient as a whole and not as someone having an isolated disease.

Those physicians interested in studying age related medicine can do so by attending courses offered by various organization across the globe with predominant being The American Academy of Anti-Aging Medicine or A4M. It is not yet formally accredited by the American Medical Association. Some scientists and physicians take offense at the name Anti-Aging as they think it is unscientific. They have suggested terms such as Age Management Medicine, Advanced Preventive Medicine, Interventive Biogerontology. But the underlying principles remain the same.

### What causes aging?

Aging is a progressive failure of metabolic processes. There is a concept of pause as put forth by Dr. Eric Braverman indicating that every organ ages at a different rate. To a certain extent it is based on hormones. All these have been put forth into various theories as follows:

1. Free Radical Theory
2. The Nuroendocrine Theory
3. Telomerase Theory of Aging
4. The Wear and Tear Theory
5. The Rate of Living Theory
6. The Waste Product Accumulation Theory
7. The Cross-linking Theory
8. The Immune Theory
9. Theories of Errors and Repairs
10. The Order to Disorder Theory

Some of these contradict and others overlap. But underlying these are three main biochemical processes involved in aging. These are oxidation, glycation and methylation. Other relevant processes are chronic inflammation and hormonal deregulation.

### Oxidation

Free radicals are a group of simple compounds with an electron missing from their chemical structure. This makes them unstable. They seek out other chemical structures from which they can acquire an electron and in the process make them unstable.

Free radicals in small and controlled quantities are useful in everyday metabolism. They take part in several normal reactions within the body including breathing. These free radicals are mainly produced during oxygen metabolism within the cells. The problem starts when the production of these free radicals increases and goes out of control.

Free radical reactions can be divided into three stages: Initiation, Propagation and Termination. The defense mechanisms against these are inactivating them within the cells soon after production, removing them by scavenging antioxidants and increasing the elimination of material already damaged by free radicals.

To put it in practice means to prevent exposure to free radicals from sources such as pollution, bad diet, and smoking and take antioxidants. The term antioxidant refers to chain-breaking compound. Broadly antioxidant is any substance, that when present at low concentrations compared with those of an oxidizable substrate significantly delays or inhibits oxidation of that substrate.

Free radicals damage the cell membrane which is composed of lipids and proteins. Their interaction results in the production of the chemical Melondialdehyde which is very harmful, contributing to another important aging process called glycation.

### Glycation

When glucose molecules and other sugars such as fructose attach themselves to proteins, it is called glycation. This results in brownish discoloration of tissues. The binding of sugar to protein causes cross linking of proteins. Cross linked proteins cause more damage by reacting with free radicals and other toxins to create Advanced Glycation Endproducts (AGEs). These AGEs bind to cells at special attachment sites called RAGEs (Receptor for AGEs). These result in the production of several harmful chemicals damaging tissues.

AGEs are found in most tissues in the body and their concentration increases from the age of twenty onward.

However nutrient such as carnosine and other Anti-Aging supplements/drugs can help in breaking this process.

Unfortunately the process of glycation not only affects proteins, but also interferes with DNA. A cross linked DNA molecule is of no use at all. There are drugs being investigated which can actually break this bond between cross linked proteins which are glycated.

### Methylation

When ‘methyl groups’ are being added to different constituents of the proteins, DNA and other molecules to keep them in good, active condition, it is called methylation. This is necessary for the normal maintenance of tissues and is usually kept at a healthy levels naturally by the body. Methylation of certain parts of the DNA causes permanent switching off of unnecessary genes and saves the body from abnormal DNA division. This means that methylation of those particular sections of DNA blocks any abnormal DNA from being passed onto the future generations of cells.

Any chronic inflammation process affects methylation because the immune system, which is heavily involved in fighting inflammation, gorges itself on methyl groups, leaving nothing for other tissues of the body.

Low methylation is reflected in the increasing levels of homocysteine, which is found in chronic inflammatory processes such as lupus, heart disease and diabetes. Increased intake of methylators reduces the risk of these diseases.

### Chronic inflammation

There are scientists who believe that most age related changes in the body are due to chronic inflammation. When there is chronic inflammation, the body tissues are eaten away by toxic chemicals, resulting in dementia, thickening of the arteries, arthritis, diabetes, hormonal imbalance and so on. Taking low dose of anti-inflammatory chemicals such as Aspirin keeps age related inflammation at bay. However, this is not a widely shared opinion. A diet containing nutrients i.e. fish (contains anti-inflammatory oils), fruit and vegetables, particularly berries (which are packed full of antioxidants and inflammation-fighting flavonoids) along with supplements like alpha lipoic acid and co-enzyme Q10 are helpful.

### Hormonal deregulation

There is little doubt about the important role hormones play in our lives and in aging. The pacemakers of youth, these precious little chemicals keep us young, healthy and vibrant. In general, aging results in imbalance of hormones in the body or one can also ask “Does hormonal imbalance cause aging?” Hormones such as growth hormone, melatonin and DHEA need to be replaced or reactivated during aging to prevent the body from falling apart. It is also important to make sure that the binding sites of the hormones need to be in good working order. According to Dr. Eric Braverman, proponent of pause theory, every organ in the body ages at a different rate. The classical example of this is the menopause in females and the literature is replete with Hormone Replacement Therapy for the same. It seems prudent to replace the other hormones in physiological limits. Hormone replacement therapy is likely to play a very important role in maintaining health in time to come. It is very important here to make the distinction between the Synthetic hormones and the Bioidentical hormones which is discussed later.

### How to put anti-aging program together?

### Principles

1. Enhance health through eating a hormonally correct diet
2. Enrich body with optimum doses of proven antioxidants and nutraceuticals
3. Improve physical exercise performance, which includes aerobic, anaerobic and flexibility training
4. Replace hormones to levels to those of 20-30 years old.

### Diet

The importance of food we eat can be summarized by what Dr. Barry Sears said and I quote: “The food you eat is probably the most powerful drug you will encounter. But to use this drug correctly, you have to apply the hormonal rules about the food that haven't changed in the past 40 million years and are unlikely to change anytime soon.”

Balancing Insulin and glucagon activity is at the core of eating a hormonally correct diet. The hormonally correct diet contains 30% protein, 50% carbohydrates and 20% fat along with vitamins, minerals and drinking plenty of water which is filtered, mineralized and magnetized. Eating small meals every 4-5 hours and eating the proper ratio of proteins, carbohydrates and fat at each meal or snack can mean a difference between illness and good health.

Eating proper food for one's height, weight and body type and activity level is the foundation for a better quality of life and greater longevity.

### Exercise

Dr. Alex Lief, MD Harvard Medical School has said and I quote: “Exercise is the closest thing we have to an anti-aging pill.” Regular physical activity has been a way of life for virtually every person who has reached the age of 100 years in sound condition. Exercise is medicine. If you think that smoking is not good, the following statement will enlighten. “Not exercising has the equivalent impact on your health as smoking one and one half pack of cigarettes a day.”

### Nutraceutical supplements

These include various antioxidants, vitamins and minerals. These include but are not limited to Vitamin A, C, E, mineral selenium, glutathione, superoxide dismutase, Coenzyme Q10, Alpha Lipoic Acid and Carnosine.

### Hormones

First we have to distinguish the synthetic hormones and bioidentical hormones. The former differ in chemical structure from the later which exactly have the same structure as the hormones we have in our body. Hence they can not be patented. These are available as injection, tablets, and creams. They are derived from Yam or Soy. The goal of hormone replacement therapy is to bring them to the level of youth. Again it has to be done after complete evaluation and under the guidance of an Anti-Aging physician.

### Future of anti-aging medicine

The future will involve manipulating genes, increasing utilization of stem cells (embryonic and adult) and targeted delivery of nutrients and drugs using nanotechnology.

It is my hope that this will help you begin your educational journey in Anti-Aging Medicine.
